# Biomimetic Strategies for Climate-Adaptive Buildings: A Multi-Case Analytical Framework Linking Biological Mechanisms, Architectural Translation, and Evidence Status

**DOI:** 10.3390/biomimetics11100711

**Published:** 2026-10-06

**Authors:** Mete Mutlu Balkis, Cemil Atakara

**Affiliations:** Department of Architecture, Faculty of Fine Arts, Design and Architecture, Cyprus International University, 99258 Nicosia, Cyprus

**Keywords:** biomimicry, biomimetics, climate-responsive architecture, analytical framework, biological mechanism, architectural translation, evidence status, environmental verification, case study

## Abstract

Biomimetic architecture is increasingly discussed as a route toward climate-responsive and regenerative design, yet architectural descriptions alone cannot demonstrate thermal, ventilation, daylight, energy or microclimatic performance. This study presents the Biomimetic Climate-Adaptability Framework (BCAF) as a retrospective analytical and comparative framework rather than a performance-rating or validated design-support instrument. The BCAF establishes an explicit chain between an environmental problem, a biological function, a biological mechanism, an architectural translation, a design parameter, an expected environmental response and an appropriate verification method. A structured qualitative multi-case analysis is applied to four cases—the Lotus Temple in New Delhi, National Taichung Theater in Taichung, Little Island in New York and Waterloo International Terminal in London—selected through transparent criteria concerning documented nature-related references, architectural translations, environmental relevance, scale diversity and source availability. The analysis distinguishes documented architectural mechanisms from verified environmental outcomes and does not convert qualitative interpretation into numerical performance scores. Cross-case comparison shows that the analytical value of the precedents lies primarily in the traceability of mechanism translation and in making evidence gaps explicit. The study contributes a mechanism-based analytical structure for structured precedent analysis and verification planning; its prospective efficacy as a design-support tool remains to be tested through expert, interrater and design action studies.

## 1. Introduction

Climate instability, urban heat stress and the need to reduce operational energy demand have increased interest in architectural strategies that adapt to environmental conditions through form, envelope, structure, material and landscape. Biomimetic approaches are relevant to this discussion because biological systems offer examples of thermoregulation, solar control, airflow management, water management, structural efficiency and ecosystem interaction [1,2,3,4,5]. However, a recurrent problem in architectural biomimicry is the tendency to move too quickly from a nature-inspired form to claims of environmental performance.

A roof profile, opening pattern, vegetated surface, water feature or articulated envelope may indicate a plausible passive mechanism, but these features are not in themselves evidence that a building achieves improved thermal comfort, ventilation, daylight, energy use or microclimatic performance. Such outcomes require validation through suitable evidence such as monitored environmental data, calibrated simulation, energy records, post-occupancy assessment, occupant surveys or controlled comparison. The distinction between design intent, plausible mechanism and verified outcome is therefore central to rigorous biomimetic research.

The present study develops the Biomimetic Climate-Adaptability Framework (BCAF) as a retrospective analytical and comparative framework rather than a numerical performance-ranking system or a prospectively validated design-support tool. Its purpose is to trace how a defined environmental problem is connected to a biological function and mechanism, how that mechanism is translated architecturally, which design parameters embody the translation, what environmental response is expected, what evidence is currently available, and how the response would need to be verified.

The study is guided by three research questions:

RQ1. How can a biomimetic architectural framework make the chain from environmental problem to biological mechanism and architectural translation explicit without conflating design evidence with measured performance?

RQ2. How do the four selected cases differ in the clarity and traceability of their biomimetic mechanism-to-architecture translation and in the evidence available for environmental claims?

RQ3. What transferable analytical principles, evidence limitations and verification requirements can be derived from the cross-case analysis for climate-responsive architectural practice?

The article first develops an analytical literature base covering biomimetic terminology, biologically inspired design processes, architectural translation and environmental performance evidence. It then explains the BCAF and transparent case-selection procedure, applies the framework to the four cases, and synthesizes implications for design and later performance verification.

## 2. Literature Review

### 2.1. Biomimicry, Biomimetics and Levels of Biological Translation

Biomimetics and biomimicry describe related approaches to learning from biological systems, although their disciplinary emphases differ. Benyus framed biomimicry through nature as model, measure and mentor [1], while Vincent et al. emphasized systematic transfer from biology to technology [2]. ISO 18458:2015 further formalizes terminology, concepts and a biomimetic development process, reinforcing the importance of a traceable relationship between biological knowledge and technical translation [3]. In architecture, Zari's organism-, behavior- and ecosystem-level distinction has been particularly influential because it differentiates imitation of biological form from emulation of processes and ecological relationships [4,5].

This distinction matters for climate-responsive design. Organism-level resemblance may carry cultural, structural or spatial value, but environmental relevance becomes stronger when a project identifies a biological function—such as regulation, protection, exchange, buffering or resource cycling—and translates the underlying mechanism into architectural parameters. The analytical task is therefore not simply to ask what natural object a building resembles, but what environmental problem is addressed, what biological function is relevant, how that function operates, and which architectural decisions attempt to reproduce the mechanism.

### 2.2. From Biological Analogy to Mechanism-Based Design

Research on biologically inspired design has repeatedly shown that successful transfer depends on abstraction rather than literal copying. Helms, Vattam and Goel identify recurring problem-driven and solution-driven pathways in biologically inspired design and also note common errors that arise when biological analogies are transferred without adequate abstraction [6]. Architectural research similarly emphasizes investigative tools, adaptive envelopes, material behavior and functional translation rather than formal resemblance alone [7,8,9,10,11,12,13,14,15,16]. Fayemi et al. further characterize biomimetics as a structured process involving abstraction, transfer tools and interdisciplinary practice, reinforcing the need to make the transition from biological knowledge to technical design explicit [17].

For architectural use, this suggests a sequence in which the designer first defines an environmental challenge, identifies a biological function relevant to that challenge, abstracts the operative mechanism, translates it into an architectural form or system, and then defines parameters that can be tested. This sequence also prevents biomimetic claims from remaining metaphorical. A biological analogy becomes methodologically useful only when the mechanism and architectural consequences are sufficiently explicit to be examined, compared and—where performance is claimed—verified.

### 2.3. Climate-Responsive Architecture and the Evidence Required for Performance Claims

Climate-responsive architecture uses passive, active or hybrid strategies to mediate heat, solar radiation, daylight, airflow, moisture and outdoor microclimates. Relevant architectural responses include orientation, massing, shading, natural ventilation, thermal mass, evaporative cooling, adaptive façades, vegetation and blue-green systems [10,11,12,13,18,19,20,21,22,23]. Yet the presence of such strategies should be distinguished from their demonstrated effectiveness. The performance of natural ventilation, daylight, thermal comfort or energy use depends on climatic boundary conditions, geometry, operation, occupancy and control; consequently, environmental claims require evidence appropriate to the outcome being asserted. Reviews of biomimetic adaptive façades likewise emphasize that early-stage design frameworks must be paired with explicit performance assessment, testing and monitoring strategies if environmental effectiveness is to be claimed [24].

The present study therefore distinguishes four types of evidence: (a) design/documentary evidence, showing that a mechanism is intentionally embodied in a project; (b) model- or simulation-based evidence, estimating the environmental response under defined assumptions; (c) monitored or operational evidence, documenting real environmental or energy outcomes; and (d) post-occupancy or user evidence, assessing the experienced conditions. These evidence types are complementary rather than interchangeable. A documented passive mechanism can support an early-stage design inference, but it cannot be reported as verified performance unless suitable performance evidence is available.

### 2.4. Research Gap and Contribution of the BCAF

The existing literature provides strong foundations for biomimetic terminology, levels of emulation, biologically inspired design processes, adaptive envelopes and climate-responsive strategies [1,2,3,4,5,6,7,8,9,10,11,12,13,14,15,16,17,18,19,20,21,22,23,24]. Reviews also show a growing body of biomimetic architectural cases [25,26,27]. The methodological need addressed here is a compact architectural analysis structure that makes the mechanism chain and the status of evidence explicit while avoiding conversion of qualitative interpretation into pseudo-quantitative performance scores.

Accordingly, the BCAF does not rank buildings by environmental performance. In the present study, it structures retrospective comparative case reading by asking seven linked questions: What environmental problem is addressed? What biological function is relevant? What mechanism performs that function? How is the mechanism translated architecturally? Which design parameters embody the translation? What environmental response is expected? What verification method would be necessary to substantiate that response? The contribution of the framework lies in integrating these questions into a single traceable architectural workflow and explicitly separating the expected response from the verified outcome. The present article demonstrates this analytical use only; potential prospective design-support use is treated as a hypothesis for later validation rather than as an established capability.

## 3. Materials and Methods

### 3.1. Research Design

The study uses a structured qualitative comparative multi-case design. Its objective is not to calculate or rank actual building performance, but to examine how biomimetic reasoning is documented and translated into architectural mechanisms across different scales and contexts. The evidence base consists of peer-reviewed literature, books, official project documentation and published architectural descriptions. No human participants, surveys, interviews, student data or experimental procedures are involved.

The procedure has five stages: (1) analytical review of the biomimetic design and environmental performance literature; (2) purposive selection of four cases using explicit inclusion criteria; (3) tracing of each case through the seven-step BCAF chain; (4) systematic classification of the available evidence supporting each environmental interpretation; and (5) cross-case synthesis of transferable analytical principles, evidence gaps and appropriate verification pathways. The method does not use numerical scoring or total rankings.

### 3.2. Case Selection and Scope

Four cases were selected purposively rather than statistically: Lotus Temple, National Taichung Theater, Little Island and Waterloo International Terminal. Inclusion required: (i) a documented nature-related or biomimetic reference; (ii) a visible translation into architectural form, spatial organization, structure, envelope or landscape; (iii) relevance to at least one climate-related design issue such as solar control, airflow, daylight, heat mitigation or microclimate; (iv) sufficient published or official documentation for mechanism tracing; and (v) contribution to diversity of scale and biomimetic level. Projects were excluded when the nature reference was purely decorative, when no architectural mechanism could be identified, or when available documentation was insufficient to distinguish design intent from later interpretation. The cases constitute a deliberately heterogeneous purposive case set rather than a controlled comparative benchmark. Their variation in typology, scale, climatic context, construction period and mode of biomimetic translation is used to examine whether the same mechanism-tracing workflow can be applied consistently across contrasting architectural conditions. Accordingly, cross-case synthesis concerns the traceability of biological-to-architectural translation and the associated evidence requirements, rather than comparative environmental performance or statistical representativeness. The resulting case-selection logic is summarized in Table 1.

### 3.3. Biomimetic Climate-Adaptability Framework (BCAF)

**Figure 1:**
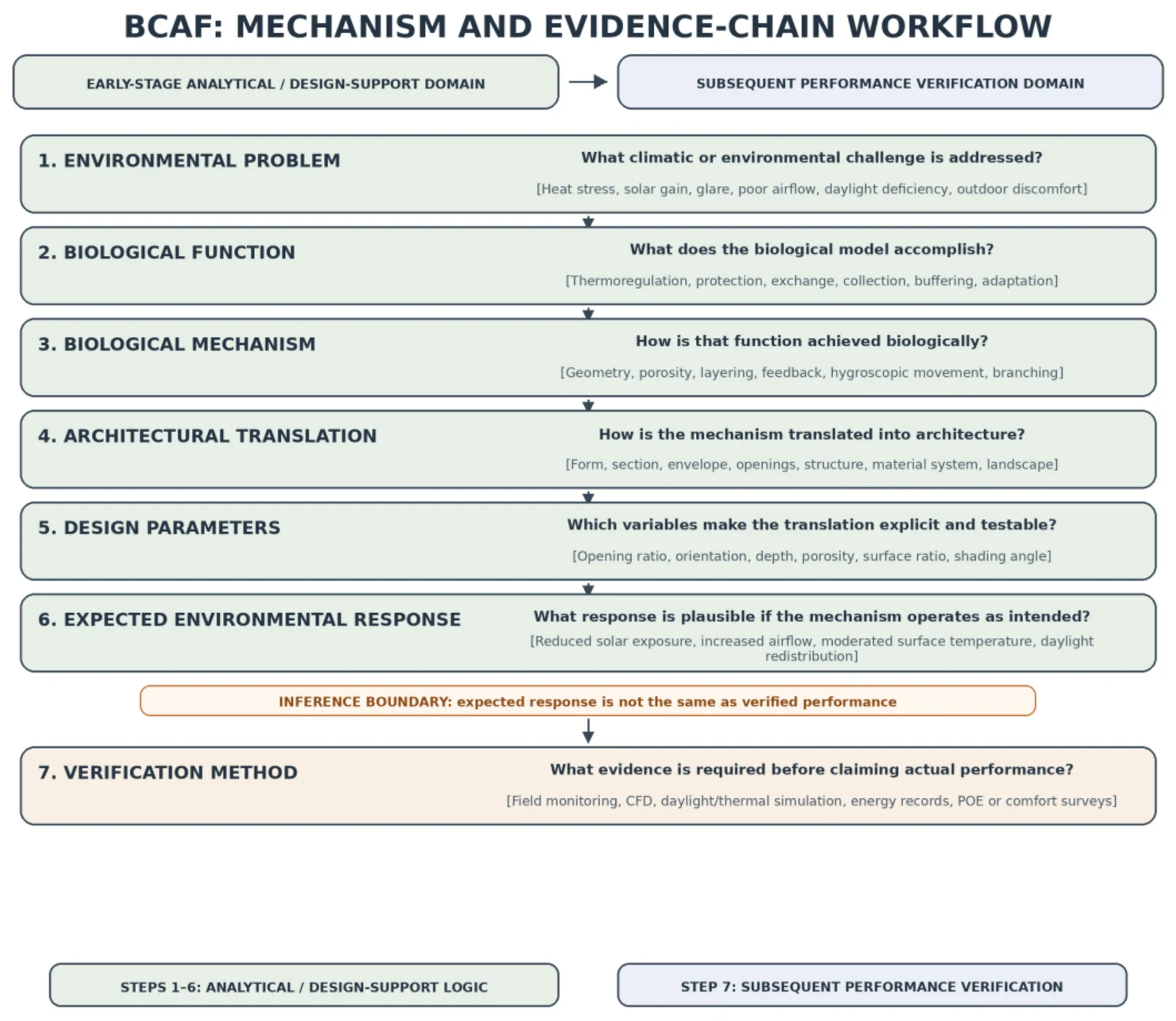
BCAF mechanism and evidence-chain workflow.

The BCAF is defined here as a mechanism-based analytical framework that makes the reasoning chain explicit and records the type and status of evidence available at each stage. It is not a scored performance evaluation instrument and, in this retrospective study, is not claimed to be a prospectively validated design-support tool. The framework consists of seven linked elements. Figure 1 summarizes the mechanism and evidence-chain workflow, while Table 2 presents the same chain in tabular form.

### 3.4. Evidence Status and Analytical Safeguards

For each case, environmental statements are reported using a standardized evidence-status notation. DE (documented evidence) indicates that the nature-related reference, architectural translation and/or intended mechanism is supported by project or scholarly documentation. ER (expected response) indicates a plausible environmental consequence derived from established passive-design principles but not verified for the specific project. VO (verified outcome) is reserved for project-specific monitoring, simulation, operational data or post-occupancy evidence. A case may therefore be coded DE + ER when its architectural translation is documented but its environmental performance remains unverified. Where the biological function or biological mechanism is not explicitly established in the reviewed sources, this absence is stated rather than inferred. For readability and methodological transparency, the cross-case application is divided into two complementary matrices presented in Section 4.5: the first reports environmental problem, biological function, biological mechanism and architectural translation, while the second reports design parameters, expected environmental response, evidence status and required verification for the corresponding case/mechanism entries.

## 4. Multi-Case Application of the BCAF

### 4.1. Lotus Temple: From Lotus Morphology to Passive Spatial Hypotheses

The Lotus Temple provides a useful test of the distinction between biological symbolism and functional translation. Its lotus-derived geometry is clearly documented at the organism level, while the large central hall, openings and surrounding pools create architectural conditions that have been associated with daylight, air movement and evaporative moderation [28]. Under the BCAF, these features are treated as design mechanisms and hypotheses rather than proof of measured environmental performance. The environmental problem may be framed as heat and daylight management in a hot climate; the relevant biological/architectural logic is interpreted through layered petal geometry, vertical spatial organization, openings and water-adjacent surfaces. Expected responses include shading, air exchange and local cooling potential. However, claims regarding thermal comfort, ventilation rates or energy reduction would require project-specific monitoring, CFD/thermal modeling or operational energy data. The case therefore demonstrates how a strong symbolic reference can be extended into a mechanism-based design reading without overstating evidence.

### 4.2. National Taichung Theater: Strong Spatial Analogy, Weaker Environmental Verification

The National Taichung Theater is characterized by continuous curved surfaces, interconnected cavities and a porous cave-like spatial concept [29]. The project is valuable precisely because it tests the limits of biomimetic interpretation. The architectural translation is strong: conventional distinctions between floor, wall and ceiling are replaced by continuous spatial morphology. Yet a compelling organic analogy does not itself establish ventilation, daylight or thermal performance. Under the BCAF, the environmental problem and biological function therefore require careful separation from the architectural concept. Porosity and interconnected voids may plausibly influence air movement, daylight penetration and spatial environmental gradients, but such responses remain hypotheses unless supported by project-specific simulations or measurements. The appropriate verification pathway would include airflow analysis, illuminance/daylight assessment and monitored indoor conditions. The case shows why the framework treats formal and spatial innovation as analytically significant while resisting unsupported performance claims.

### 4.3. Little Island: Ecosystem-Oriented Landscape and Microclimate Hypotheses

Little Island extends the analysis to an urban landscape in which elevated planted topography, structural pots, vegetation and waterfront exposure operate as an integrated public-space system [30]. The relevant environmental problems include solar exposure, outdoor thermal stress, habitat provision and ecological continuity. The ecosystem-level reading is stronger than a single-organism analogy because the project combines topography, vegetation, water adjacency and public use. Expected responses may include differentiated shade, evapotranspirative cooling, wind modification, habitat support and varied outdoor comfort conditions. Nevertheless, vegetation and topography do not by themselves prove improved microclimate. Verification would require microclimatic monitoring or validated modeling of air temperature, mean radiant temperature, wind speed, surface temperature and thermal-comfort indices, ideally compared with an appropriate baseline. The case therefore illustrates how the BCAF can identify both ecosystem-oriented design logic and the evidence needed to substantiate urban-climate claims. This verification requirement is consistent with field-based urban-climate research showing that land-cover type and green infrastructure can produce measurable differences in outdoor thermal stress, but that these effects must be established through observed or modeled microclimatic variables rather than inferred from vegetation presence alone [31].

### 4.4. Waterloo International Terminal: Articulated Envelope and Testable Technical Parameters

Waterloo International Terminal offers a technical example of nature-related envelope interpretation. Its roof and glazing system respond to changing geometry, structural movement, daylight requirements and a constrained rail site; the overlapping articulated components have often been compared with scale-like protection [32,33]. The BCAF focuses less on resemblance than on the mechanism of articulated, variable enclosure. Architectural parameters include roof curvature, overlapping components, glazing geometry and structural accommodation of movement. Expected environmental responses include daylight admission and protection from external conditions, but claims concerning daylight quality, heat gain or energy performance require simulation or measured evidence. Because the envelope is materially and geometrically explicit, the case demonstrates how a biomimetic analogy can be translated into testable design parameters even where comprehensive operational environmental data are not publicly available.

### 4.5. Cross-Case Mechanism, Evidence and Verification Matrices

To preserve the seven-step BCAF logic without compressing conceptually distinct fields into a single wide matrix, the cross-case synthesis is presented in two linked tables. Table 3 focuses on the mechanism-to-architecture chain; Table 4 focuses on testable parameters, expected responses, evidence status and the verification still required. The case order is identical in both tables so that the two matrices can be read together as one complete BCAF application.

## 5. Results

### 5.1. What the Non-Scored Comparison Reveals

The analysis does not produce a league table of cases. Instead, it reveals differences in mechanism traceability and evidence status. Waterloo International Terminal provides a relatively explicit technical translation because the envelope mechanism can be described through concrete geometric and construction parameters. Little Island provides the clearest ecosystem-scale translation but requires dedicated microclimate data before climatic benefits can be quantified. The Lotus Temple combines a highly legible biological form with plausible passive mechanisms, yet measured building performance evidence is limited in the sources used here. The National Taichung Theater presents strong spatial and morphological innovation, while the connection between its biomimetic analogy and specific environmental outcomes remains comparatively interpretive.

Across all four cases, the most defensible comparison is therefore not ‘which building performs best?’ but ‘how clearly can the chain from biological reference to architectural mechanism, expected response and verification need be traced?’ This shift directly addresses the methodological risk of assigning numerical values to outcomes that have not been measured under comparable conditions.

### 5.2. Evidence Gaps Are Themselves an Analytical Result

The case analysis shows that documentation quality is uneven. Architectural publications often describe concept, form and construction more extensively than environmental monitoring. Table 3 and Table 4 make this asymmetry explicit: Table 3 separates the biological function and mechanism from their architectural translation, while Table 4 records whether the resulting environmental interpretation is documented, expected or project-specifically verified and identifies the evidence still required. A case with a compelling mechanism but limited monitoring remains valuable as an analytical precedent, but the framework prevents that precedent from being presented as verified environmental performance. Required verification is therefore part of the analytical result rather than an optional future-work note, because it specifies what evidence would be necessary to move from design hypothesis to performance claim.

## 6. Discussion

### 6.1. From Performance Scoring to Mechanism Traceability

The BCAF reports mechanism traceability without numerical aggregation. A 0–5 scale or similar arithmetic rating could imply a degree of objectivity or performance validation that is not supported when the underlying evidence consists mainly of architectural documentation and qualitative interpretation. Transparency is therefore maintained through explicit analytical questions, evidence categories and verification requirements rather than numerical scores.

This framing also clarifies the appropriate scope of the present evidence. The current study demonstrates the BCAF retrospectively as an analytical structure for mechanism traceability, evidence-status reporting and verification planning. It does not demonstrate prospective design-support efficacy. The BCAF may provide a basis for later design-stage use because its fields identify problems, mechanisms, parameters and tests, but that prospective utility requires separate validation through expert assessment, interrater testing and design action studies.

### 6.2. Theoretical Foundation and Relationship to Earlier Biomimetic Methods

The BCAF is grounded in three established strands of research: biological abstraction and function transfer [2,3,6,17]; architectural levels of biomimicry and ecosystem thinking [4,5]; and climate-responsive/adaptive-envelope research [7,8,9,10,11,12,13,15,16,17,18,19,20,21,22,23,24]. Its contribution is not a new biological taxonomy and it is not presented as a replacement for established biomimetic design methods. Rather, the BCAF occupies a narrower analytical niche by combining explicit mechanism-to-architecture traceability with design parameters, evidence-status reporting and a required verification pathway. Table 5 positions these features relative to selected established approaches.

The comparison is deliberately limited to methodological emphases that are directly relevant to this article. It does not rank the approaches or imply that the BCAF supersedes them; instead, it clarifies the narrower analytical gap addressed by the BCAF.

### 6.3. Transferability and Limits

The transferability identified in this retrospective analysis should be understood at the level of mechanism rather than copied form. For example, the analytical lesson from an articulated envelope is not to reproduce its appearance but to identify the environmental or geometric problem, understand the operative principle and specify the parameters that would need recalibration for another climate, program, material system or construction context. Likewise, ecosystem-oriented landscape strategies require recalibration to local climate, species, maintenance regimes and urban morphology. Any prospective design application of these analytical lessons remains subject to validation.

## 7. Analytical Implications and Prospective Design Relevance

• In retrospective analysis, define the environmental problem explicitly before interpreting a biological reference.

• Report biological function and biological mechanism separately from visual resemblance; where either is not documented, state the gap.

• Distinguish architectural translation from the design parameters through which the translation could be tested.

• State environmental consequences as expected responses unless project-specific evidence verifies them.

• Link each expected environmental response to an appropriate verification method; for example, airflow claims should indicate CFD or air-speed/ACH measurement, and microclimate claims should indicate appropriate monitored or simulated indicators.

• Use cross-case precedents analytically to compare mechanism traceability rather than to rank unverified performance.

• Treat absence of performance data as an evidence limitation, not as a reason to infer a positive outcome.

• For future prospective applications, the BCAF may be used to structure hypotheses before simulation or monitoring, but its efficacy as a design-support tool requires separate validation.

## 8. Limitations and Future Research

The study has four principal limitations. First, the four purposively selected cases do not represent the full field of biomimetic architecture. Second, documentation is heterogeneous and does not provide comparable measured environmental data for all cases. Third, the BCAF has not yet undergone expert panel validation or interrater reliability testing. Fourth, this article does not independently conduct CFD, energy simulation, daylight analysis, environmental monitoring or post-occupancy evaluation. Consequently, the analysis supports retrospective design interpretation, evidence classification and verification planning, not comparative claims of actual building performance or demonstrated prospective design-support efficacy. In the present study, the BCAF is demonstrated as a retrospective analytical framework rather than empirically validated as a generative design tool. Its operational efficacy in architectural studios or professional decision-making therefore remains a proposition to be tested through prospective design action studies, expert evaluation and interrater reliability assessment.

Future work should test the framework with a larger and more systematically sampled case database; use multiple independent assessors to examine coding consistency; and apply project-specific simulation, monitoring and post-occupancy methods to selected cases. A particularly useful next step would be to test whether projects with more explicit mechanism chains also produce more reliable or measurable environmental outcomes. Such research would allow the operational usefulness and performance-related validity of the BCAF to be assessed more directly.

## 9. Conclusions

This study investigates four contrasting cases of biomimetic strategies in climate-adaptive architecture through the Biomimetic Climate-Adaptability Framework. In this article, the BCAF is presented and demonstrated as a mechanism-based retrospective analytical framework rather than a numerical performance evaluation or prospectively validated design-support tool. It links environmental problems, biological functions, biological mechanisms, architectural translation, design parameters, expected environmental responses, evidence status and verification methods.

Across the four cases, the analysis indicates that architectural features can provide evidence of design intent and plausible mechanisms, but they should not be treated as proof of thermal, ventilation, daylight, energy or microclimatic performance. The revised cross-case matrix also shows that biological function and biological mechanism are not equally explicit in all published precedents; in some cases, this absence is itself an analytical finding. The BCAF therefore makes evidence limits visible and identifies the testing needed for stronger claims. Its demonstrated contribution lies in improving the traceability and methodological discipline of retrospective biomimetic case analysis, while prospective design-support efficacy remains a subject for future validation.

## Figures and Tables

**Table 1:** Case-selection logic. The cases are analytical examples rather than a statistically representative sample.

Case	Context/Designer	Primary Nature-Related Reference	Analytical Scale	Reason Retained
Lotus Temple	New Delhi/Fariborz Sahba	Lotus geometry; central hall, openings and water setting	Organism + behavior	Examines the transition from symbolic biological form to plausible passive spatial mechanisms.
National Taichung Theater	Taichung/Toyo Ito & Associates	Porous, cave-like continuous spatial morphology	Form + spatial behavior	Examines whether a strong organic/spatial analogy can be distinguished from substantiated climate performance.
Little Island	New York/Heatherwick Studio + MNLA	Elevated planted topography and ecosystem-like landscape relationships	Ecosystem/urban landscape	Extends analysis from building systems to landscape, habitat and microclimate-related design intent.
Waterloo International Terminal	London/Grimshaw	Overlapping scale-like roof/envelope articulation	Behavior + envelope	Examines technical translation of adaptive/protective logic into structure, glazing, curvature and daylight-related design.

**Table 2:** BCAF mechanism chain.

BCAF Element	Analytical Question	Typical Evidence/Output
1. Environmental problem	What climatic or environmental challenge is being addressed?	Heat stress, solar gain, glare, poor airflow, daylight deficiency, outdoor discomfort, ecological loss.
2. Biological function	What does the biological model accomplish?	Regulation, protection, exchange, movement, collection, buffering, adaptation, resource cycling.
3. Biological mechanism	How is that function achieved biologically?	Geometry, porosity, layering, feedback, hygroscopic movement, branching, evaporative process, distributed networks.
4. Architectural translation	How is the mechanism converted into architecture?	Form, section, envelope, openings, structure, material system, landscape or water system.
5. Design parameter	Which variables make the translation testable?	Opening area, orientation, depth, porosity, surface ratio, canopy density, shading angle, section height, material response.
6. Expected environmental response	What response is plausible if the mechanism operates as intended?	Reduced solar exposure, increased airflow, moderated surface temperature, daylight redistribution, local shading, habitat support.
7. Verification method	What evidence is required before claiming actual performance?	Field monitoring, CFD, daylight/thermal simulation, energy records, controlled baseline comparison, POE or comfort survey.

**Table 3:** Cross-case BCAF mechanism-to-architecture matrix. Biological function, biological mechanism and architectural translation are reported as separate fields. Where the reviewed project documentation does not establish a biological environmental function or a transferred biological mechanism, the absence is stated rather than inferred.

Case	Environmental Problem	Biological Function	Biological Mechanism	Architectural Translation
Lotus Temple	Heat, glare, daylight and airflow management	Not explicitly established as a biological environmental function in the reviewed project sources; lotus morphology is the documented nature reference.	Not explicitly established as a transferred biological mechanism in the reviewed project sources; layered petal morphology is the documented organism-level reference.	Layered petal-like shell geometry, tall central volume, openings and water-adjacent setting.
National Taichung Theater	Daylight and environmental distribution in deep public interiors	Not explicitly documented as a biological function; spatial exchange/porosity is an analytical environmental reading.	No specific biological mechanism is explicitly documented in the reviewed project sources; the cave-like/organic continuity functions as a spatial analogy.	Continuous curved surfaces, interconnected cavities and distributed apertures.
Little Island	Outdoor heat stress, habitat and public-space microclimate	Exposure buffering, habitat support and environmental exchange at ecosystem level.	Distributed canopy shade, evapotranspiration, vegetation-water interaction and topographic modulation.	Elevated planted topography, varied canopy, water-edge landscape and structural pots.
Waterloo International Terminal	Daylight, enclosure and geometric/site adaptation	Protection with flexibility and controlled environmental exposure.	Overlapping articulated scale-like elements providing continuous protection while accommodating local geometric variation and movement.	Articulated overlapping roof and glazing components adapted to changing curvature and structural movement.

**Table 4:** Cross-case BCAF evidence-status and verification matrix. DE = documented evidence; ER = expected response; VO = verified project-specific outcome. The table reports the evidence level and the required verification for each corresponding case/mechanism entry and does not treat an expected response as verified performance.

Case	Design Parameters	Expected Environmental Response	Evidence Status	Required Verification
Lotus Temple	Opening position/area; section height; shell/shading geometry; water proximity	Air movement, shading, daylight modulation and local evaporative moderation	DE + ER; project-specific VO for thermal, airflow or energy performance not identified in the reviewed sources.	CFD; thermal monitoring; illuminance/daylight measurement; operational energy data; baseline comparison.
National Taichung Theater	Void connectivity; aperture distribution; depth; surface geometry	Potential airflow and daylight redistribution	DE for architectural/spatial translation + ER; biological mechanism remains partly interpretive; project-specific VO not identified in the reviewed sources.	CFD; daylight simulation/measurement; monitored indoor conditions; controlled baseline where feasible.
Little Island	Canopy density; species/LAI; shade coverage; topographic height; wind exposure	Shade, evapotranspiration, habitat support and differentiated outdoor comfort	DE + ER; project-specific microclimate VO not identified in the reviewed sources.	Microclimate monitoring or validated modeling; air/MRT/surface temperature; wind; PET/UTCI; biodiversity survey.
Waterloo International Terminal	Glazing ratio; curvature; overlap; orientation; structural movement allowance	Daylight admission and protective envelope response	DE + ER; comprehensive project-specific environmental VO not identified in the reviewed sources.	Daylight/solar simulation; temperature/energy monitoring; baseline comparison.

**Table 5:** Methodological positioning of the BCAF relative to selected established biomimetic approaches. The comparison identifies differences in emphasis rather than ranking the approaches.

Approach	Primary Purpose	Function/Mechanism Abstraction	Architectural Translation	Evidence-status Distinction	Performance Verification Planning
ISO 18458:2015 [3]	Domain-general biomimetic terminology and development process	Explicit biological-to-technical abstraction/transfer	General technical transfer; not architecture-specific	Not focused on architectural evidence-status reporting	Verification depends on application domain; not an architectural performance matrix
Helms et al. [6]	Problem-driven and solution-driven biologically inspired design process	Strong emphasis on abstraction and transfer from biology	Technical design translation	Not a primary focus of the cited process model	Testing may occur in design development, but evidence-status classification is not the model's central purpose
Zari [4,5]	Levels of biomimicry and ecosystem-oriented sustainability	Distinguishes organism, behavior and ecosystem levels	Strong architectural/ecosystem interpretation	Does not primarily classify project-specific performance evidence	Performance verification is not structured as the terminal step of the level taxonomy
Badarnah & Kadri [14]	Generation of biomimetic design concepts	Function-based biological exploration and concept generation	Explicit architectural concept translation	Not the primary emphasis of the methodology	Environmental testing can follow concept generation but is not formalized as an evidence-status field
Fayemi et al. [17]	Biomimetic process, tools and interdisciplinary practice	Structured abstraction and transfer	General technical translation across disciplines	Evidence status is not architecture-specific	Evaluation is process-dependent rather than an explicit architectural verification column
BCAF (present study)	Retrospective mechanism-traceability and evidence analysis	Separates biological function and biological mechanism	Separates architectural translation from explicit design parameters	Explicit DE/ER/VO reporting for each analyzed case	Verification method is required as part of the analytical chain; prospective design use remains unvalidated

## Data Availability

No new empirical data were generated or analyzed during the current study. The case-based information synthesized in this article is derived from publicly available published sources and project documentation cited in the manuscript. Data sharing is not applicable to this article.
